# Liposomal Glutathione Helps to Mitigate *Mycobacterium tuberculosis* Infection in the Lungs

**DOI:** 10.3390/antiox11040673

**Published:** 2022-03-30

**Authors:** Nala Kachour, Abrianna Beever, James Owens, Ruoqiong Cao, Afsal Kolloli, Ranjeet Kumar, Kayvan Sasaninia, Charles Vaughn, Mohkam Singh, Edward Truong, Christopher Khatchadourian, Christina Sisliyan, Klara Zakery, Wael Khamas, Selvakumar Subbian, Vishwanath Venketaraman

**Affiliations:** 1 Graduate College of Biomedical Sciences, Western University of Health Sciences, Pomona, CA 91766, USA; nala.kachour@westernu.edu (N.K.); abrianna.zorzi@westernu.edu (A.B.); kayvan.sasaninia@westernu.edu (K.S.); charles.vaughn@westernu.edu (C.V.); mohkam.singh@westernu.edu (M.S.); edward.truong@westernu.edu (E.T.); 2 College of Osteopathic Medicine of the Pacific, Western University of Health Sciences, Pomona, CA 91766, USA; james.owens@westernu.edu (J.O.); ruoqiong.cao@bcm.edu (R.C.); chris.khatchadourian@westernu.edu (C.K.); christina.sisliyan@westernu.edu (C.S.); klara.zakery@westernu.edu (K.Z.); 3 Public Health Research Institute Center, New Jersey Medical School, Rutgers University, Newark, NJ 07103, USA; ak1482@njms.rutgers.edu (A.K.); rk879@njms.rutgers.edu (R.K.); subbiase@njms.rutgers.edu (S.S.); 4 College of Veterinary Medicine, Western University of Health Sciences, Pomona, CA 91766, USA; wkhamas@westernu.edu

**Keywords:** glutathione, tuberculosis, antioxidant, reactive oxygen species, cytokine, granuloma

## Abstract

*Mycobacterium tuberculosis* (*M. tb*), the causative agent of tuberculosis (TB), is responsible for causing significant morbidity and mortality, especially among individuals with compromised immune systems. We have previously shown that the supplementation of liposomal glutathione (L-GSH) reduces *M. tb* viability and enhances a Th-1 cytokine response, promoting granuloma formation in human peripheral blood mononuclear cells in vitro. However, the effects of L-GSH supplementation in modulating the immune responses in the lungs during an active *M. tb* infection have yet to be explored. In this article, we report the effects of L-GSH supplementation during an active *M. tb* infection in a mouse model of pulmonary infection. We determine the total GSH levels, malondialdehyde (MDA) levels, cytokine profiles, granuloma formation, and *M. tb* burden in untreated and L-GSH-treated mice over time. In 40 mM L-GSH-supplemented mice, an increase in the total GSH levels was observed in the lungs. When compared to untreated mice, the treatment of *M. tb*-infected mice with 40 mM and 80 mM L-GSH resulted in a reduction in MDA levels in the lungs. L-GSH treatment also resulted in a significant increase in the levels of IL-12, IFN-γ, IL-2, IL-17, and TNF-α in the lungs, while down-regulating the production of IL-6, IL-10, and TGF-β in the lungs. A reduction in *M. tb* survival along with a decrease in granuloma size in the lungs of *M. tb*-infected mice was observed after L-GSH treatment. Our results show that the supplementation of mice with L-GSH led to increased levels of total GSH, which is associated with reduced oxidative stress, increased levels of granuloma-promoting cytokines, and decreased *M. tb* burden in the lung. These results illustrate how GSH can help mitigate *M. tb* infection and provide an insight into future therapeutic interventions.

## 1. Introduction

According to the World Health Organization (WHO), a quarter of the world’s population has asymptomatic, latent *Mycobacterium tuberculosis* (*M. tb*) infection (LTBI) [1]. *M. tb*, the causative agent of tuberculosis (TB), is an intracellular bacterial pathogen that primarily inhabits the lower respiratory tract. In addition to symptomatic, active TB, the reactivation of LTBI can also be detrimental to the host and may contribute to the transmission of the disease [2]. Conventional anti-TB therapies, involving multiple antibiotics administered for a prolonged time (minimum of six months for drug-sensitive TB), are hindered by poor compliance rates of patients and undesired side effects [3]. In addition, the development of multi-drug-resistant strains of *M. tb* rendered traditional therapies ineffective, prompting the necessity for new therapeutic development [4].

*M. tb* sequestration and the inhibition of phagocytic maturation pathways in host macrophages leads to persistent stimulation of host-immune cells leading to LTBI [2,5]. In individuals with LTBI, mycobacterial growth and dissemination are inhibited by a localized proinflammatory response at the site of infection. Macrophages and other immune cells, such as neutrophils, dendritic cells, natural killer cells, fibroblasts, and T cells, are recruited by a Th-1 cytokine-mediated response to the site of infection, allowing for the encapsulation of *M. tb* within granulomas [6,7]. The physical barrier created by granuloma, coupled with a lack of oxygen availability at the center of the granuloma, induces a non-replicating state of *M. tb* [8]. This, in turn, leads to an inhibition of mycobacterial growth and a localized site for continuous T-cell activation [9]. The cytokine response to *M. tb* varies depending on the progression of the infection and whether it is active or latent, but it is well established that Th-1 cytokines, such as TNF-α and IFN-γ, are principally important for granuloma formation and maintenance. Furthermore, IL-17 is associated with protection against TB and a strong immune response to TB infection [10,11]. The most common route of *M. tb* reactivation is through granuloma liquefaction as a result of necrosis and caseation [12]. Increased caseation results in the cavitation of the granulomas, and leads to *M. tb* dissemination and systemic spread of infection [13].

Oxidative stress is implicated in the pathogenesis of LTBI reactivation [14,15,16]. Immunocompromised individuals have higher levels of oxidative stress and are at an increased risk of LTBI reactivation [17,18]. Such patients have been observed to have a dysregulated cytokine response and impaired granuloma formation, coupled with increased levels of lipid peroxidation by-products and diminished synthesis of glutathione (GSH)-producing enzymes [19,20,21]. GSH is an endogenous antioxidant tripeptide consisting of glutamine, cysteine, and glycine. The antioxidant property of reduced GSH can prevent immune cell damage caused by reactive oxygen species (ROS). The supplementation of an oral liposomal formulation of GSH (L-GSH), which allows for the intracellular delivery of GSH, has been observed to decrease oxidative stress in HIV and T2DM patients, both immunocompromised populations [22,23]. Aside from serving a protective role against oxidative damage by ROS, L-GSH supplementation in patients with HIV or T2DM was associated with an induction of a Th-1 cytokine response, improved granuloma formation of peripheral blood mononuclear cells infected with *M. tb*, and a decrease in the burden of *M. tb* in vitro [23,24,25].

Given the potential immunomodulatory and antimycobacterial effects of GSH revealed in previous studies, we aim to assess the immunomodulatory effects of L-GSH supplementation on the host response to *M. tb* infection in mouse lungs in vivo. We determine the levels of oxidative stress and GSH in untreated and L-GSH-treated mice. We examine the levels of TNF-α, IL-12, IFN-γ, IL-2, TGF-β, IL-10, IL-17, and IL-6 in lung lysates obtained from *M. tb*-infected mice at different timepoints post-infection. In addition to *M. tb* survival in the lungs, we also evaluate the involvement of the lungs in granuloma formation.

## 2. Materials and Methods

### 2.1. Bacteria Preparation

Virulent *Mycobacterium tuberculosis* strain H37Rv (ATCC, Manassas, VA, USA) was propagated in Middlebrook 7H9 media supplemented with 0.05% Tween-80 and oleic acid albumin dextrose catalase (OADC) mix (Thermo Fisher Scientific, Waltham, MA, USA), as previously described [26]. Briefly, the H37Rv culture was grown until it reached the logarithmic phase, with an absorption value (OD_600_) between 0.6 to 0.8. The mycobacterial culture was harvested by centrifugation at 3000× *g* rpm, and the bacterial pellet was gently vortexed with 3 mm sterile glass beads to break down the bacterial clumps. Larger clumps were filtered by a 5 µm syringe filter. The bacterial concentration was determined by a CFU assay [26]. Briefly, serial dilutions of bacterial culture were plated on Middlebrook 7H10 agar plates and incubated at 37 °C with 5% CO_2_ for 3–4 weeks. The number of bacterial CFU was enumerated, and mycobacterial cultures were aliquoted in 1 mL cryotubes and stored at −80 °C until ready to use. All general chemicals and reagents were purchased from Millipore-Sigma, St. Louis, MO, USA, unless otherwise specified.

### 2.2. Study Design and Mice Information

**Figure 1:**
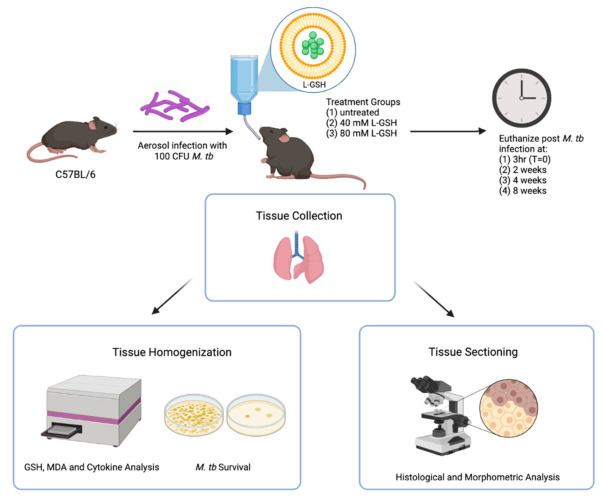
Schema of the study design. The C57BL/6 mice were infected with 100 CFU H37Rv *M. tb* strain. The mice were treated with either 40 mM L-GSH, 80 mM L-GSH, or untreated before euthanizing at 3 h (T = 0), 2-, 4-, and 8-weeks post-infection. The lungs were collected and either homogenized or sectioned for GSH, MDA, and cytokine analysis, or histological and morphometric analysis, respectively. The sample size (n) includes 4 mice in the 3 h group and 6 mice, each for the 2, 4, and 8 weeks groups. The figure was created using biorender.com.

Specific pathogen-free (SPF) male and female C57BL/6 wild-type (WT) mice of 8–10 weeks old were purchased from Envigo (Envigo, Indianapolis, IN, USA) and used for *M. tb* infection in biocontainment level 3 (BSL3) settings, as previously described [27]. Preliminary studies conducted in our lab suggest that 40 mM and 80 mM L-GSH concentrations should be sufficient for increasing the GSH levels in C57BL/6 WT mice (unpublished). Accordingly, L-GSH at 40 mM or 80 mM was administered to mice daily in the drinking water, starting on the day of infection until the experimental endpoint (up to 8 weeks post-infection) (Figure 1). L-GSH was provided by our collaborator, Dr. Guilford, from Your Energy Systems, Palo Alto, CA. USA. The L-GSH dissolved in water has a fresh, refreshing taste with a hint of a sweet smell, and our previous studies have shown that mice drank this solution without any hesitation at an average of 2 mL per day per mouse, similar to plain water without L-GSH [22,24]; there was no difference between male and female mice in their water intake. We also included an untreated but *M. tb* infected group as our experiment control. The untreated group was only given water. All the water or supplemental drinking was replaced every three days. Each experimental group consisted of 6 mice, 3 males and 3 females (for T = 0 (i.e, 3 h post-infection), 2 males and 2 females were used). Our prior studies in the mice model of *M. tb* infection show that a minimum of 4 animals per group is required to have enough statistical power to differentiate between groups [27]. Mice were infected with approximately 100 CFU of *M. tb* H37Rv via the Glas-Col aerosolization apparatus (Glas-Col, Terre Haute, IN, U.S.A). Mice were euthanized at 3 h, 2 weeks, 4 weeks, and 8 weeks post-infection to determine the extent of *M. tb* growth, and the structure of granulomas and the changes in the levels of GSH, malondialdehyde (MDA), and cytokines in the lungs. The time points were chosen according to the growth curve of *M. tb* in mice [28]. Lungs samples were collected at the time of necropsy, and the right superior lobe, middle lobe, post-caval lobe, and a portion of the left lung were homogenized to analyze the bacterial load (see below) and relevant biomarkers, including GSH, MDA, and pro- and anti-inflammatory cytokine levels. A portion of the left lung lobe was fixed in neutral formalin for histologic analysis. Histology was performed to observe granuloma structures and/or tissue necrosis within the lungs.

### 2.3. Ethics Statement

All animal experiments were conducted humanely in accordance with the procedures of NIH guidance. All animal protocols were approved by the Institutional Animal Care and Use Committee (IACUC) of the Western University of Health Sciences and the Rutgers University New Jersey Medical School (Protocol#20190007).

### 2.4. Assay of the MDA Levels in the Lung Lysates

The MDA levels in the infected lung homogenates were measured at 3 h, 2 weeks, 4 weeks, and 8 weeks post-infection. The MDA measurements were performed using the TBARS assay kit (Cayman Chemical Company, Ann Arbor, MI, USA, catalog #10009055). The MDA levels were measured colorimetrically at 530–540 nm as per the procedures included in the kit. The amount of MDA was normalized for the total protein levels from each sample, which were measured using the bicinchoninic acid (BCA) assay kit (catalog #23227) as per the instructions of the manufacturer (Thermo Fisher Scientific, Waltham, MA, USA).

### 2.5. Assay of the GSH Levels in the Lung Lysates

The total GSH was measured in the mice lung lysates using the GSH colorimetric detection kit (Thermo Fisher Scientific Waltham, MA, USA, catalog #EIAGSHC). Ice cold 5% sulfosalicylic acid was used to remove the protein present in the samples. The GSH concentrations were measured as an endpoint read of the color developed at 405 nm. The concentrations of GSH were normalized to the total protein concentrations of each sample. The total protein levels were quantified using the BCA assay kit.

### 2.6. Quantification of the Cytokines in the Lung Lysates by ELISA

Homogenized mouse lungs at 3 h, 2 weeks, 4 weeks, and 8 weeks post-infection were used for the cytokine measurements. The cytokine levels were measured using the following ELISA kits: IL-6 (catalog #88-7064-88), IFN-γ (catalog #88-7314-88), IL-2 (catalog #88-7024-88), IL-10 (catalog #88-7105-88), TGF-β (catalog #88-8350-88), TNF-α, IL-12 (catalog #BMS6004), and IL-17 (catalog #88-7002-88), as per the manufacturer’s instructions (Thermo Fisher Scientific, Waltham, MA, USA).

### 2.7. Bacterial CFU Assay

The bacterial load in mice organs was determined by CFU assay, as we previously reported [29]. Briefly, portions of the lung from the 3 groups of mice (untreated, 40 mM GSH, and 80 mM GSH) at 3 h, 2 weeks, 4 weeks, and 8 weeks post-infection were homogenized and used for the CFU assay. The tissue samples were homogenized in 2 mL of sterile 1X PBS, serially diluted in 1XPBS and plated on Middlebrook 7H11 agar supplemented with OADC (Thermo Fisher Scientific, Waltham, MA, U.S.A). The inoculated agar plates were incubated at 37 °C for 3–4 weeks for colonies to appear. The final CFU load in the lungs was then calculated per mL of the lysate.

### 2.8. Histological Analysis

Formalin-fixed lung samples were embedded in paraffin wax and sectioned into a thickness of 5 microns using a microtome (Veterinary Pathology Center at Western University of Health Sciences) and placed on glass slides.

Hematoxylin and eosin (H&E) (Poly Scientific, Bay Shore, NY, USA) staining was performed on the sectioned lungs to visualize the distribution and organization of the host cells. Deparaffinized lung sections were rehydrated by serial treatment with absolute and 95% ethanol before soaking with distilled water. Slides were first stained with hematoxylin and rinsed with water before eosin staining. Slides were dehydrated in serial treatment of 95% ethanol, absolute ethanol, and xylene before coverslip mounting. A morphometric analysis of mouse lung granulomas was performed, as previously described [30], using PathScan Enabler (Meyer Instruments, Houston, TX, USA) and SigmaScan Pro software (Systat Software Inc., San Jose, CA, USA). The extent of fibrosis and tissue modeling in the lungs of L-GSH-treated and -untreated mice was evaluated at 4- and 8-weeks post-infection by Masson’s trichrome staining procedure, as previously described [31]. The slides were microscopically analyzed by two investigators with one of them blindfolded to the sample identity. The opinion of the investigators was consistent with the observation of the histologic changes in mice lungs.

### 2.9. Statistical Analysis

The GraphPad Prism Software 8 was utilized for statistical analysis. One-way ANOVA with Tukey’s post hoc correction was performed when comparing more than two categorical variables. The unpaired *t*-test with Welch corrections was performed when comparing the two categories. All reported values represent the mean +/− standard deviation values for each category, and a *p*-value of <0.05 was considered significant. Any placement of an asterisk (*) denotes a direct comparison to the previous category. ** indicates a significant difference with a *p*-value < 0.005.

## 3. Results

### 3.1. Levels of MDA in the Lungs of Untreated and L-GSH-Treated Mice Infected with M. tb

**Figure 2:**
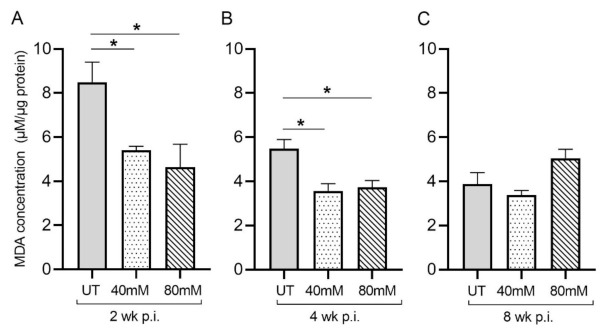
L-GSH treatment significantly reduced the malondialdehyde (MDA) levels in the lungs of *M. tb*-infected mice at 2 weeks and 4 weeks post-infection. The MDA levels in the lung lysates of untreated (UT) or 40 mM or 80 mM L-GSH-treated *M. tb*-infected mice were measured spectrophotometrically. The lung lysates were collected from the untreated mice and mice treated with 40 mM or 80 mM L-GSH at 2 weeks (**A**), 4 weeks (**B**), and 8 weeks (**C**) post-infection (p.i.). All of the values plotted for each group represent the mean values +/− standard deviation. One-way ANOVA with Tukey’s multiple group post correction was applied to determine the significance between groups, and a *p*-value of <0.05 (*) was considered significant. Statistical analysis was performed using GraphPad Prism Software 8. The sample size is 6 mice (n = 6) per group, per time point.

**Figure 3:**
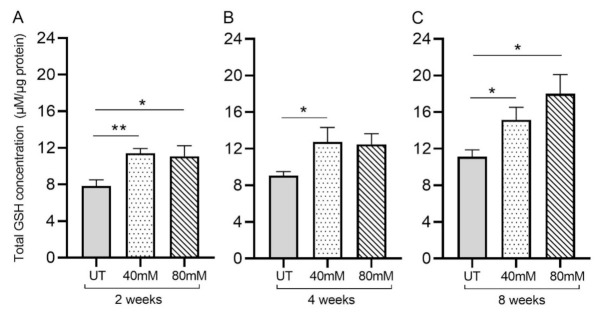
L-GSH supplementation significantly increased the total GSH levels in the *M. tb*-infected mice lungs. The total GSH levels in the lungs were measured spectrophotometrically. The lung lysates were collected from mice treated with 40 mM or 80 mM L-GSH or untreated mice at 2 weeks (**A**), 4 weeks (**B**), and 8 weeks (**C**) post-infection (p.i.). All of the values plotted for each group represent the mean values +/− standard deviation. One-way ANOVA with Tukey’s multiple group post-correction was applied to determine the significance between groups, and a *p*-value of <0.05 (*) was considered significant. ** *p* < 0.01. Statistical analysis was performed using GraphPad Prism Software 8. The sample size is 6 mice (n = 6) per group, per time point.

**Figure 4:**
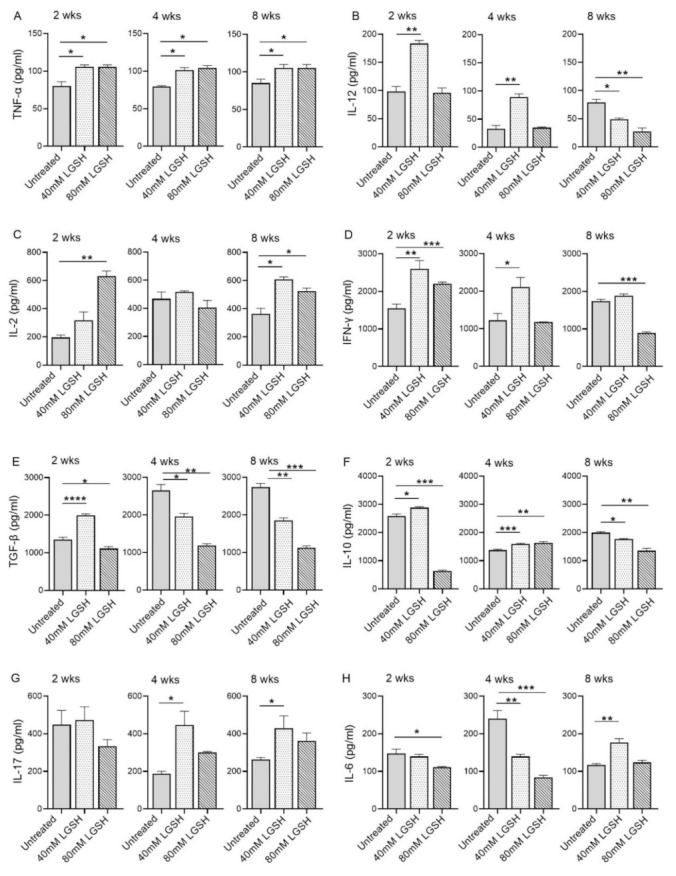
L-GSH treatment significantly affected the production of pro- and anti-inflammatory cytokines in the *M. tb*-infected mice lungs. The proinflammatory cytokines TNF-α (**A**), IL-12 (**B**), IL-2 (**C**), IFN-γ (**D**) and anti-inflammatory molecules TGF-β (**E**), IL-10 (**F**), as well as the IL-17 (**G**) and IL-6 (**H**) levels were measured in the lung lysates of the *M. tb*-infected untreated mice and the 40 mM or 80 mM L-GSH-treated mice at 2 weeks, 4 weeks, and 8 weeks post-infection. All of the values plotted for each group represent the mean values +/− standard deviation. One-way ANOVA with Tukey’s multiple group post correction was applied to determine the significance between the groups and a *p*-value of <0.05 (*) was considered significant. ** *p* < 0.01; *** *p* < 0.001; **** *p* < 0.0001 Statistical analysis was performed using GraphPad Prism Software 8. The sample size is 6 mice (n = 4) per group, per time point.

Malondialdehyde (MDA) is a by-product of lipid peroxidation and serves as a proxy for measuring oxidative stress due to free-radical production. In *M. tb*-infected mice treated with L-GSH at 40 mM and 80 mM, the MDA levels in the lung decreased significantly, compared to the untreated groups, at 2 weeks (Figure 2A) and 4 weeks (Figure 2B) post-infection (Figure 3). Although the levels of MDA in L-GSH-treated mice lungs were similar between 4- and 8-weeks post-infection, the untreated animals also had reduced MDA levels, comparable to the L-GSH-treated animals (Figure 2C). No significant difference in the MDA levels was noted at 8 weeks post-infection between L-GSH-treated and -untreated mice lungs. These results suggest that L-GSH treatment dampened the MDA levels, although no concentration-dependent response was noted between 40 mM and 80 mM L-GSH treatment groups.

### 3.2. GSH Levels in the Lungs of Untreated and L-GSH-Treated Mice Infected with M. tb

Treatment of *M. tb*-infected mice with L-GSH resulted in a significant increase in the total GSH in the lung lysates at 2-, 4-, and 8-weeks post-infection, compared to the untreated group (Figure 3A–C). Although treatment with 80 mM L-GSH resulted in an increase in the total form of GSH in the lung lysates at 4 weeks post-infection, which is comparable to the 40 mM L-GSH treatment group, the levels were not statistically significant compared to the untreated group (Figure 3B). Our results indicate that treatment with 40 mM L-GSH is sufficient to significantly elevate the levels of total GSH in the lungs.

### 3.3. Measurement of the Cytokine Levels in the Lungs of Untreated and L-GSH-Treated Mice Infected with M. tb

Subsequently, we investigated the level of key proinflammatory and anti-inflammatory cytokines in the lungs of the *M. tb*-infected mice with or without the L-GSH treatment at 2, 4, and 8 weeks post infection (Figure 4). The level of TNF-a was significantly increased in both the 40 mM and 80 mM L-GSH-treated animals, compared to the untreated group, at 2-, 4-, and 8-weeks post-infection (Figure 4A). Although the level of IL-12 was elevated in the untreated group at 2 weeks post-infection, the treatment with 40 mM L-GSH significantly increased the levels of this cytokine further (Figure 4B). Similarly, a significant increase in IL-12 was noted in the 40 mM L-GSH-treated animals at 4 weeks post-infection. However, the levels of IL-12 were significantly reduced in both the 40 mM and 80 mM L-GSH-treated animals, compared to the untreated mice at 8 weeks post-infection.

The levels of IL-2 were increased in the 40 mM and 80 mM L-GSH-treated animals, compared to the untreated group, at 2- and 8-weeks post-infection, although a significant difference was noted only for the 80 mM L-GSH treatment at 2 weeks. (Figure 4C). The levels of IL-12 were similar and comparable between the untreated and L-GSH-treated animals at 4 weeks post-infection. This observation suggests a concentration-dependent increase in the IL-2 levels between the 40 mM and 80 mM L-GSH supplementation at the early stages of infection (2 weeks). Further, the effect of the L-GSH supplementation appears to be dependent on specific infection stages.

The treatment of the *M. tb*-infected mice with 40 mM and 80 mM L-GSH resulted in a statistically significant increase in the levels of IFN-γ at 2 weeks post-infection (Figure 4D). However, at 4- and 8-weeks post-infection, a significant increase in the IFN-γ levels was noted only in the 40 mM L-GSH. Indeed, the *M. tb*-infected mice treated with 80 mM L-GSH showed a reduction in IFN-γ levels, compared to the 40 mM L-GSH treatment, suggesting that the latter dose is more effective in elevating the levels of IFN-γ.

The level of TGF-β in the lungs of the untreated mice gradually increased from 2 weeks until 4 weeks and remained at similar levels between 4- and 8-weeks post-infection (Figure 4E). A transient but significant increase in the TGF-β levels was noted at 2 weeks post-infection in the mice treated with 40 mM L-GSH, compared to the untreated animals. However, compared to the untreated group, the treatment with 40 mM and 80 mM L-GSH significantly decreased the lung TGF-β levels at both 4- and 8-weeks post-infection.

Similar to TGF-β, the level of IL-10 was significantly increased at 2 weeks post-infection in mice treated with 40 mM L-GSH, while the level of IL-10 was significantly reduced in 80 mM L-GSH-treated animals, compared to the untreated group (Figure 4F). However, treatment with either 40 mM or 80 mM significantly increased the level of IL-10 at 4 weeks, before the levels were significantly reduced at 8 weeks post-infection in the L-GSH-treated animals, compared to the untreated controls.

No significant change in the IL-17 levels was noted between the untreated and L-GSH-treated mouse lung homogenates at 2 weeks post-infection (Figure 4G). However, compared to the untreated, L-GSH treatment increased the levels of IL-17 at 4 and 8 weeks post-infection, although the difference was statistically significant only in 40 mM L-GSH-treated animals

The level of IL-6 was comparable between the untreated and 40 mM L-GSH-treated animals at 2 weeks post-infection, although treatment with 80 mM L-GSH significantly reduced the IL-6 levels (Figure 4H). At 4 weeks post-infection, the IL-6 levels were significantly reduced in both 40 mM and 80 mM L-GSH-treated animals, compared to the untreated controls. However, a significantly increased IL-6 level was noted between the untreated and 40 mM L-GSH-treated animals at 8 weeks post-infection.

Taken together, the levels of various pro- and anti-inflammatory cytokines suggest the differential effect of L-GSH between early/acute (2 weeks) and late/chronic (8 weeks) stages of infection, and that L-GSH supplementation may have opposite effects, depending on specific disease stages. These results indicate that most of the host-beneficial responses, mediated by the proinflammatory responses against *M. tb* infection, were exerted by 40 mM L-GSH supplementation.

### 3.4. M. tb Survival in the Lungs of Untreated and L-GSH-Treated Mice

**Figure 5:**
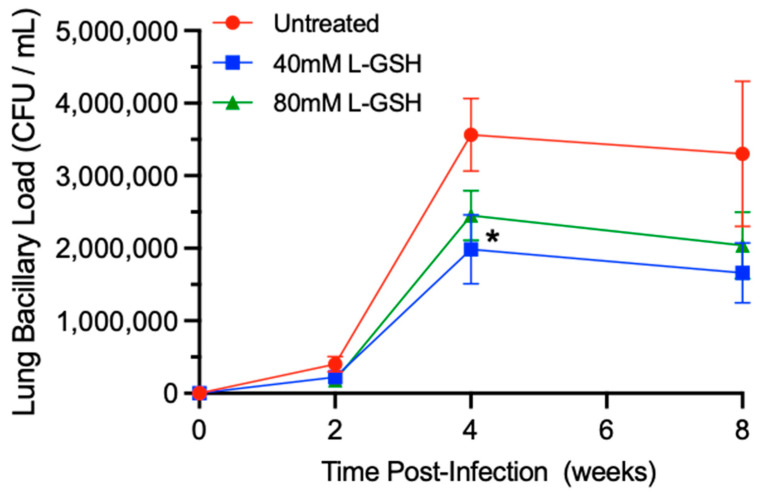
L-GSH supplementation reduced the survival of *M. tb* in the lungs of the L-GSH-treated mice. *M. tb* survival was quantified in the lung homogenates of untreated (UT) and the 40 mM or 80 mM L-GSH-treated mice at 2 weeks, 4 weeks, and 8 weeks post-infection. The bacterial CFU assay was performed to determine the bacterial survival in the lungs. All of the values plotted for each group represent the mean values +/− standard deviation. One-way ANOVA with Tukey’s multiple group post correction was applied to determine the significance between the groups and a *p*-value of <0.05 (*) was considered significant. Statistical analysis was performed using GraphPad Prism Software 8. The sample size is 6 mice (n = 6) per group, per time point.

To determine the effect of L-GSH supplementation on the bacterial load in *M. tb*-infected mice, we examined the number of bacterial colony forming units (CFUs) in the lysates of the lungs (Figure 5). When compared to untreated mice, the treatment of *M. tb*-infected mice with L-GSH resulted in a reduction in the bacterial load in the lungs at 2-, 4-, and 8-weeks post-infection, although the difference was significant only in 40 mM L-GSH-treated animals at 4 weeks post-infection (Figure 5).

Thus, treatment with L-GSH, particularly at a 40 mM concentration, reduces the *M. tb* burden in the lungs compared to the untreated animals.

### 3.5. Morphometric Analysis of Lung Granulomas in the M. tb-Infected Mice with or without L-GSH Treatment

**Figure 6:**
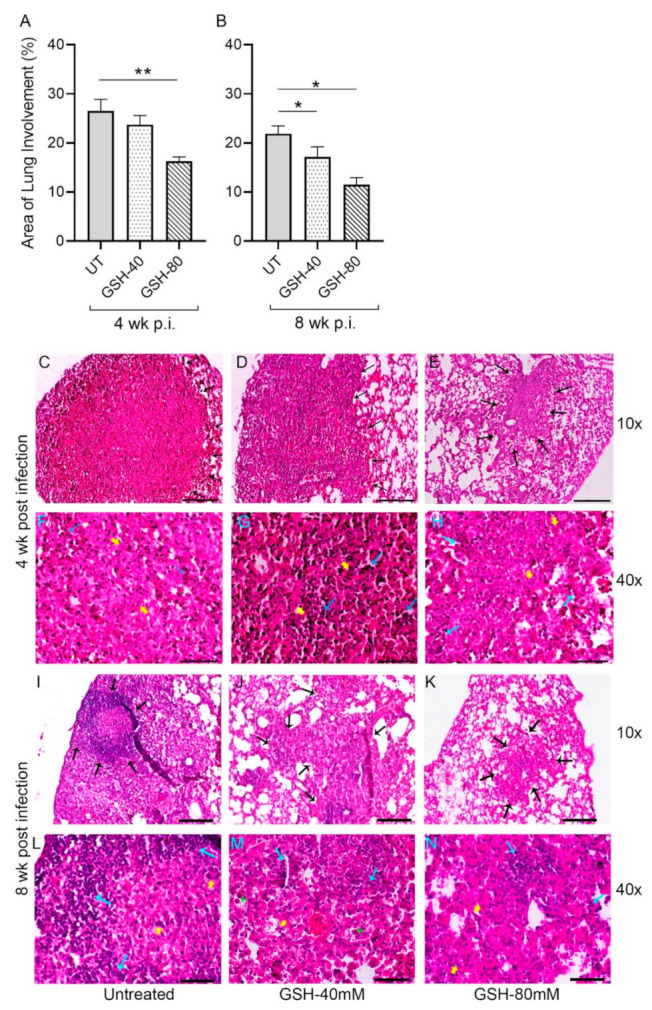
Morphometric analysis and histology of the lung granulomas in the *M. tb*-infected untreated and L-GSH-treated mice. The lung sections were collected from the *M. tb*-infected untreated mice and the 40 mM or 80 mM L-GSH-treated mice at 4 weeks (**A**) and 8 weeks (**B**) post-infection (p.i). All of the values plotted for each group represent the mean values +/− standard deviation. One-way ANOVA with Tukey’s multiple group post correction was applied to determine the significance between the groups and a *p*-value of <0.05 (*) was considered significant. ** *p* < 0.01. Statistical analysis was performed using GraphPad Prism Software 8. The sample size is 6 mice (n = 6) per group, per time point. The lung histopathology was analyzed from the untreated and 40 mM or 80 mM L-GSH-treated mice at 4 weeks (**C**–**H**) and 8 weeks (**I**–**N**) post-*M. tb* infection (p.i.). The lung sections were stained with hematoxylin and eosin to visualize the presence of granulomas, inflammation, cell infiltration, and cellular aggregates with and without L-GSH treatment under bright field microscopy. The images were magnified at 10× (**C**–**E**,**I**–**K**) and 40× (**F**–**H**,**L**–**N**). The lung samples of the untreated (**C**,**F**,**I**,**L**) and 40 mM (**D**,**G**,**J**,**M**) or 80 mM (**I**,**H**,**K**,**N**) L-GSH-treated mice were analyzed blind-folded. The yellow arrows indicate the MNGCs, the black arrows demarcate the granulomatous area, blue arrows indicate lymphocytic cellular infiltrates, and green arrows (M) show neutrophils. The scale bars in (**C**–**E**,**I**–**K**) are 100 µm, and the scale bars in (**F**–**H**,**L**–**N**) are 50 µm. The images are representative of n = 6 per group, per time point.

**Figure A1:**
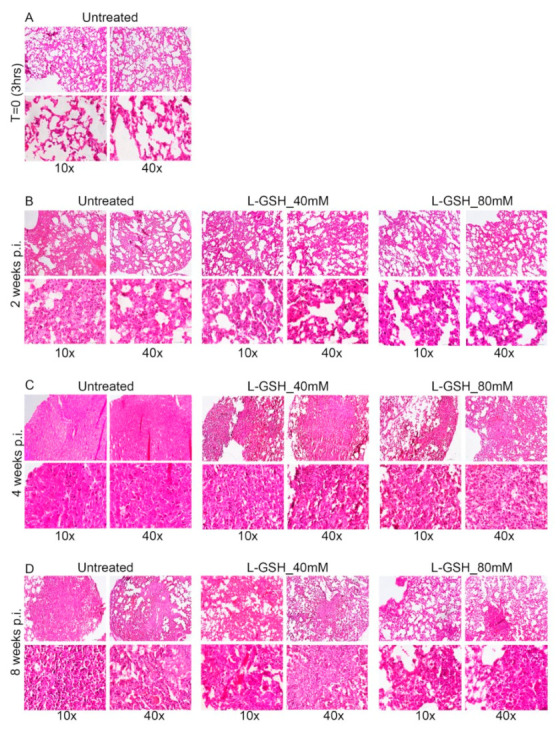
Additional images of the lung granuloma histology in *M. tb*-infected untreated and L-GSH-treated mice (40 mM and 80 mM L-GSH treatment). The images are of mice euthanized at 3 h post-infection (**A**), 2 weeks post-infection (**B**), 4 weeks post-infection (**C**), and 8 weeks post-infection (**D**). The lung sections were stained with hematoxylin and eosin to visualize the presence of granulomas, inflammation, cell infiltration, and cellular aggregates with and without L-GSH treatment under bright field microscopy. The images are magnified at 10× and 40×.

Sections of the lung from multiple *M. tb*-infected mice with or without L-GSH treatment were morphometrically analyzed for granuloma size at 4 weeks (Figure 6A) and 8 weeks (Figure 6B) post-infection (also see Figure A1). The area of the lung involved in granuloma formation was reduced in L-GSH-treated animals, compared to the untreated mice, at 4- and 8-weeks post-infection; although, the difference was statistically significant between the untreated and 80 mM L-GSH-treated animals at 4 weeks (Figure 6A). At 8 weeks post-infection, both 40 mM and 80 mM L-GSH treatment significantly reduced the area of granuloma, compared to the untreated mice lungs (Figure 6B). Together, the morphometric data suggest that L-GSH treatment reduces the lung area involved in granuloma formation in *M. tb*-infected animals.

Topologically, large, well-formed granulomas with vast immune cell infiltration and inflammation were observed in the lung sections of *M. tb*-infected mice at 4 weeks post-infection, without any treatment (Figure 6C,F). Multinucleated giant cells (MNGCs) were present, and lymphocyte aggregates were found within the granulomatous area. However, the *M. tb*-infected mice treated with 40 mM GSH showed relatively smaller, mildly inflammatory granulomas, compared to the untreated group (Figure 6D,G). These granulomas had more lymphocytic infiltration and few MNGCs. Furthermore, compared to the *M. tb*-infected untreated animals, those mice treated with 80 mM GSH had very small lesions with minimal inflammation. These granulomas had fewer lymphocyte aggregates and MNGCs (Figure 6E,H).

The architecture of the lung granulomas in *M. tb*-infected mice with or without GSH treatment at 8 weeks post-infection closely resembled that observed at 4 weeks. However, the untreated animals had lymphocyte-rich cuffs surrounding the central core of the granulomas (Figure 6I,L), while 40 mM L-GSH-treated animals showed smaller granulomas with a localized aggregation of lymphocytes (Figure 6J,M). *M. tb*-infected mice treated with 80 mM L-GSH had the least disease pathology in the lungs, with much smaller granulomas, compared to the untreated animals (Figure 6K,N). These results are consistent with the morphometric analysis and illustrate that L-GSH treatment reduces the granuloma size and complexity in *M. tb*-infected animals.

**Figure A2:**
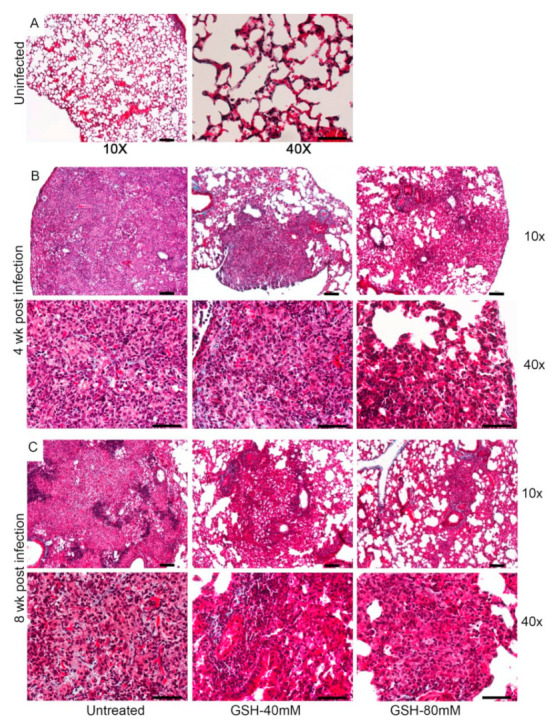
Images of the lung granuloma histology in *M. tb*-infected untreated and L-GSH-treated mice (40 mM and 80 mM L-GSH treatment). The images are of mice euthanized at 3 h post-infection (**A**), 4 weeks post-infection (**B**), and 8 weeks post-infection (**C**). The lung sections were stained with Masson’s trichrome staining to visualize the fibrosis and collagen deposition. The images are magnified at 10× and 40×.

To determine the effect of L-GSH treatment on the tissue remodeling in the lungs of M.tb-infected mice, we performed Masson’s trichrome staining of the lung sections and analyzed for fibrosis and collagen deposition. As shown in Figure A2, mild fibrosis is noted in all the infected animals at 4 and 8 weeks post-infection, and no striking differences in fibrosis and/or collagen deposition can be observed between the untreated and L-GSH-treated mice lungs (Figure A2).

## 4. Discussion

Glutathione (GSH) is an endogenous tripeptide antioxidant that serves an essential role in maintaining host redox homeostasis and preventing damage to the immune cells by toxic reactive oxygen species (ROS) [32]. Mounting evidence suggests that supplementation with liposomal GSH (L-GSH)—enhancing intracellular GSH delivery—improves *M. tb* control [33]. L-GSH was previously observed to exhibit both immunomodulatory and direct antimycobacterial effects against *M. tb* in vitro. The exogenous addition of GSH was found to enhance the functions of natural killer cells to inhibit the growth of *M. tb* in human monocytes [34]. A previous study involving mouse macrophages demonstrated that GSH is directly toxic to mycobacteria, although the mechanism of action is still not clearly understood [35]. Our lab found that a diethyl maleate-induced depletion of GSH during an active *M. tb* infection in a mouse model led to increased oxidative stress, a dysregulated immune response, and increased *M. tb* growth and burden [36]. Altogether, these studies implicate the potential role of GSH in controlling *M. tb* growth and L-GSH serving as an adjunctive therapeutic candidate for the treatment of *M. tb* infection. However, there has yet to be a study that assesses the effects of L-GSH treatment during an active *M. tb* infection in vivo. We are the first to test the effects of L-GSH supplementation in alleviating oxidative stress and improving host immune responses against an active *M. tb* infection in the lung parenchyma of C57BL/6 WT mice.

GSH levels were assessed in untreated *M. tb*-infected mice to monitor the effect of *M. tb* on levels of GSH over time. Previous findings demonstrate that GSH levels are diminished over the course of *M. tb* infection due to increases in oxidative stress brought on by the infection [37]. This phenomenon was observed in our results. We observed an increase in oxidative stress during *M. tb* infection in untreated mice that was accompanied by a diminishment in the levels of total GSH in the lungs; compared to the untreated *M. tb*-infected mice at the 3 h time point, MDA levels, indicative of oxidative stress, increased, while levels of GSH decreased at 2 weeks post-infection (not shown). This decrease in GSH can lead to a buildup of tissue-damaging ROS, leading to further complications of *M. tb* infection. While the mechanism behind this GSH diminishment is not clearly understood, it is known that, as the *M. tb* infection worsens, ROS levels increase in proportion, and the antioxidant capabilities of GSH become taxed. The antioxidant abilities of L-GSH are illustrated in our results as *M. tb*-infected mice treated with 40 mM and 80 mM L-GSH experienced a reduction in the levels of MDA in the lungs, indicating a reduction in oxidative stress compared to untreated mice at 2 weeks and 4 weeks post-infection (Figure 2A,B). When compared to the untreated *M. tb*-infected mice, treatment with 40 mM L-GSH resulted in a significant increase in the levels of total GSH in the lung at 2 weeks, 4 weeks, and 8 weeks post-infection (Figure 3A–C). Treatment of *M. tb*-infected mice with 80 mM L-GSH also resulted in a significant increase in the levels of total GSH in the lung lysates at 2 weeks and 8 weeks post-infection (Figure 3A,C), further emphasizing that L-GSH supplementation aids in the maintenance of GSH levels in the lungs and diminishes oxidative stress.

A hallmark of latent *M. tb* infection (LTBI) is characterized by the presence of granulomas [38]. Granulomas are complex cellular structures that form to inhibit the growth and dissemination of an intracellular pathogen by forming an aggregate of immune cells around the infected cells [38,39]. The recruitment and encapsulation of infected cells within a granuloma are coordinated by a T-helper-1 (Th1) cytokine response mediated by T-helper cells [40]. Cytokines, such as IFN-γ and TNF-α, facilitate the formation and maintenance of granulomas; IFN-γ also enhances the macrophage function against the mycobacterium by increasing the levels of nitrosoglutathione (GSNO) [41,42]. IL-2 stimulates T-cell differentiation as well as enhances T-cell viability. IL-12, also a polarizing cytokine, induces CD4 T cells to differentiate into a Th1 subset, which, in turn, produces IL-2 and IFN-γ [43,44,45]. IL-17 is also known for upregulating the immune response, providing protection against *M. tb* infection [46,47]. IL-10 and TGF-β, produced by macrophages and regulatory T cells, are immunosuppressive cytokines, and an overproduction results in decreased granulomatous responses [48,49]. IL-6 is an inflammatory cytokine that has been implicated in the induction of oxidative stress and can therefore be used as an oxidative stress marker [50]. Because it causes oxidative stress, increased IL-6 levels are usually associated with a decrease in GSH levels. In recent studies, L-GSH has been shown to enhance the adaptive immune response by maintaining the viability and functions of CD4+ and CD8+ T cells within infected granulomas, decreasing the expression of the programmed death receptor 1 (PD-1), increasing autophagy, and stimulating an increase in the production of IFN-γ and TNF-α [12]. Therefore, we monitored the levels of IL-6, IFN-γ, IL-2, IL-10, TGF-β, TNF-α, IL-12, and IL-17 in the lung lysates of *M. tb*-infected mice with and without L-GSH treatment at varying stages of the infection to assess the granulomatous response.

Compared to the untreated controls, mice supplemented with L-GSH had significantly decreased levels of IL-6, IL-10, and TGF-β, and increased levels of IFN-γ, IL-2, TNF-α, IL-12, and IL-17 in the lung lysates (Figure 4). These results show that supplementation with 40 mM and 80 mM L-GSH increased the expression of granuloma-supportive cytokines (IFN-γ, IL-2, TNF-α, IL-12, and IL-17), which are indicative of a stronger immune response against *M. tb* infection. Importantly, recall that the promotion of increased IFN-γ and TNF-α allows for the formation of a more robust granulomatous response and an enhancement of the macrophage effector function to control the intracellular *M. tb* infection [51]. Again, these findings implicate that supplementation with L-GSH could help to control *M. tb* infection by increasing protective cytokines and decreasing immunosuppressive cytokines.

The supplementation of L-GSH has been previously shown to significantly decrease *M. tb* survival in the peripheral blood mononuclear cells (PBMCs) of individuals infected with HIV and in human monocyte-derived macrophages (HMDMs) of people with T2DM [25,42]. In our current study, a reduction in *M. tb* survival was observed in the lungs of mice with 40 mM L-GSH treatment, with a significant decrease in *M. tb* burden at 4 weeks post-infection (Figure 5).

Decreases in mycobacterial burden are associated with a reduction in fibrotic granulomas in macaques [39,52]. Granulomas, while serving a protective role, can paradoxically contribute to pulmonary disease as macrophages in the granuloma undergo differentiation, promoting fibrogenesis and necrosis during *M. tb* infection [53]. Excessive collagen production from macrophage-derived myofibroblasts, in addition to the fusion of macrophages into multinucleated giant cells (MNGCs), can serve to isolate *M. tb* at the site of infection [54,55]. Nevertheless, fibrosis also limits the flexibility of the lungs during inspiration, induces dyspnea, and reduces lung function [54]. The size and complexity of granulomas can signify the trajectory and severity of restrictive lung disease.

However, although the mice supplemented with L-GSH had a reduced lung area involved in granuloma formation and fewer MNGCs compared to the untreated groups (Figure 6), no striking differences in fibrosis were noted from Masson’s trichome staining (Figure A2). This observation is expected, since mice are known to not undergo caseous necrosis in the *M. tb*-infected granuloma [56,57]. This is a major reason why TB is not deadly in mice. We expect that if such a large area of granuloma formation was to be observed in humans, much more fibrosis would be detected, though further studies using human samples are needed.

The relatively smaller granulomas in the L-GSH-treated mice were also found to have more localized lymphocyte aggregates. Coupled with a decreased mycobacterial burden, smaller granulomas after L-GSH treatment indicate improved granulomatous control of *M. tb* infection. We also expect that the observed reduced granuloma size, along with a decrease in the pro-fibrogenic cytokine TGF-β after L-GSH treatment, could potentially mitigate the extent of restrictive pulmonary fibrotic diseases after an *M. tb* infection [39,58] in humans; again, further studies using human samples are required to suggest this. Our results indicate that the supplementation with L-GSH leads to the formation of a smaller, more robust granuloma containing and minimizing *M. tb* growth and protecting the lungs from oxidative damage by ROS.

A differential response between 40 mM and 80 mM of L-GSH treatment has been noted, as the 40 mM L-GSH treatment appeared to exhibit an optimal granulomatous response to *M. tb* infection compared to the 80 mM L-GSH treatment, suggesting a non-monotonic dose–response curve. At most time points, treatment with 40 mM L-GSH led to increased GSH levels; decreased oxidative stress; an induction of a Th1 cytokine response with increases in IFN-γ, IL-2, and IL-12; decreased immunosuppressive cytokines TGF-β and IL-10; increased levels of IL-17 and TNF-α; decreased levels of IL-6; reduced *M. tb* burden; and improved granulomatous control. Treatment with 80 mM L-GSH appeared to induce a partial Th1 cytokine response with a nonsignificant reduction in *M. tb* burden in the lungs. The mechanism behind these differences is unclear; however, excess availability of thiols has been observed to affect ROS-sensitive signaling pathways involved in autophagy and apoptosis and can potentially contribute to redox imbalances affecting immunity and inflammation, though further studies are needed [59]. The limitations of our study include the use of a mouse model, which may lead to minor differences in the results compared to humans. Furthermore, this study did not test the effects of L-GSH supplementation during GSH deficiency, as all mice are not GSH deficient. GSH deficiency is primarily observed in immunocompromised patients (such as those with type 2 diabetes) [25] and is associated with increased susceptibility of *M. tb* infections [34,60]. Therefore, to fully assess the effects of L-GSH supplementation after GSH diminishment, we plan to conduct future studies, in which *M. tb*-infected diabetic mice (db/db) will be administered with L-GSH, which has previously been shown to work well alongside antibiotics during in vitro *M. tb* infection. The in vitro administration of L-GSH, coupled with the first-line antibiotics rifampicin, isoniazid, and pyrazinamide, led to a significant reduction in *M. tb* viability within granulomas compared to antibiotic treatment alone [12]. Therefore, our future studies will help us to understand the effects of L-GSH supplementation during immunodeficiency (and subsequently GSH-deficiency) against TB infection and understand the combined effects of L-GSH and rifampicin treatment on host immune responses in diabetes.

## 5. Conclusions

**Figure 7:**
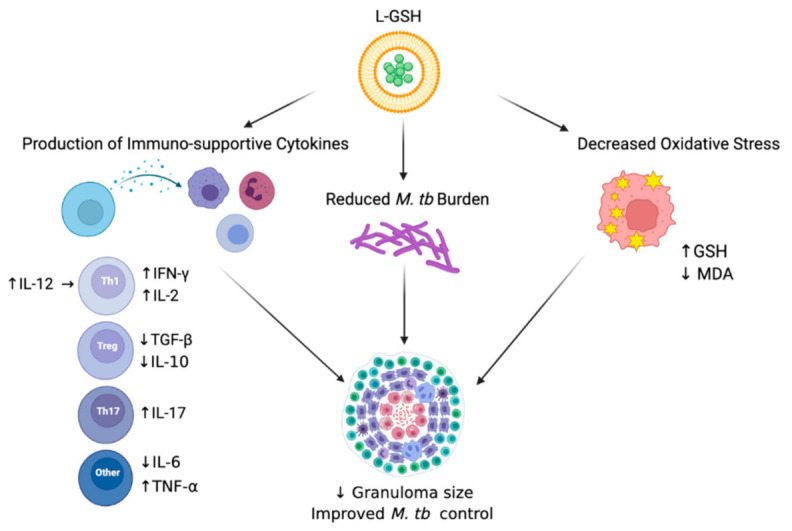
Summary of the results. Treatment of L-GSH was associated with decreased oxidative stress, the induction of a Th1 cytokine response, and reduced *M. tb* burden in infected mice organs. L-GSH treatment increased the GSH levels and decreased the levels of lipid peroxidation by-product, MDA, resulting from decreased oxidative stress. An increase in IFN-γ, TNF-α, IL-2, IL-12, and IL-17 levels was also noted after L-GSH treatment, while IL-10, TGF-β, and IL-6 levels were decreased in this group. A significant decrease in *M. tb* CFUs was observed in the lungs after L-GSH treatment. An upward arrow (↑) resembles an increase; a downward arrow (↓) resembles a reduction. The figure was created using biorender.com.

Overall, we observed beneficial host effects upon L-GSH treatment in *M. tb*-infected mice. In summary, our results illustrate that the supplementation of *M. tb*-infected WT C57BL/6 mice with 40 mM L-GSH increased the levels of immune-supportive cytokines in the lungs, reduced *M. tb* burden in the lungs, and decreased oxidative stress in the lungs, all leading to improved *M. tb* control in the lung granulomas (Figure 7). Our data suggest that L-GSH supplementation therapy may be an ideal adjunctive therapeutic candidate in mitigating *M. tb* infection.

## Data Availability

Data is contained within the article.

## References

[1] World Health Organization (2018). Latent Tuberculosis Infection: Updated and Consolidated Guidelines for Programmatic Management.

[2] Kiazyk S., Ball T. (2017). Latent tuberculosis infection: An overview. Can. Commun. Dis. Rep..

[3] Pease C., Hutton B., Yazdi F., Wolfe D., Hamel C., Quach P., Skidmore B., Moher D., Alvarez G.G. (2017). Efficacy and completion rates of rifapentine and isoniazid (3HP) compared to other treatment regimens for latent tuberculosis infection: A systematic review with network meta-analyses. BMC Infect. Dis..

[4] Seung K.J., Keshavjee S., Rich M.L. (2015). Multidrug-Resistant Tuberculosis and Extensively Drug-Resistant Tuberculosis. Cold Spring Harb. Perspect. Med..

[5] Zhai W., Wu F., Zhang Y., Fu Y., Liu Z. (2019). The Immune Escape Mechanisms of *Mycobacterium tuberculosis*. Int. J. Mol. Sci..

[6] Lyadova I.V., Panteleev A.V. (2015). Th1 and Th17 Cells in Tuberculosis: Protection, Pathology, and Biomarkers. Mediat. Inflamm..

[7] Sia J.K., Rengarajan J. (2019). Immunology of *Mycobacterium tuberculosis* Infections. Microbiol. Spectr..

[8] Russell D.G., Cardona P.J., Kim M.J., Allain S., Altare F. (2009). Foamy macrophages and the progression of the human tuberculosis granuloma. Nat. Immunol..

[9] Shkurupiy V.A., Kim L.B., Potapova O.V., Cherdantseva L.A., Putyatina A.N., Nikonova I.K. (2014). Fibrogenesis in granulomas and lung interstitium in tuberculous inflammation in mice. Bull. Exp. Biol. Med..

[10] Khader S.A., Cooper A.M. (2008). IL-23 and IL-17 in tuberculosis. Cytokine.

[11] Scriba T.J., Kalsdorf B., Abrahams D.-A., Isaacs F., Hofmeister J., Black G., Hassan H.Y., Wilkinson R., Walzl G., Gelderbloem S.J. (2008). Distinct, specific IL-17- and IL-22-producing CD4+ T cell subsets contribute to the human anti-mycobacterial immune response. J. Immunol. Baltim. Md. 1950.

[12] Lowe D.M., Redford P.S., Wilkinson R.J., O’Garra A., Martineau A.R. (2012). Neutrophils in tuberculosis: Friend or foe?. Trends Immunol..

[13] Abrahem R., Cao R., Robinson B., Munjal S., Cho T., To K., Ashley D., Hernandez J., Nguyen T., Teskey G. (2019). Elucidating the Efficacy of the Bacille Calmette-Guérin Vaccination in Conjunction with First Line Antibiotics and Liposomal Glutathione. J. Clin. Med..

[14] Venketaraman V., Millman A., Salman M., Swaminathan S., Goetz M., Lardizabal A., Hom D., Connell N.D. (2008). Glutathione levels and immune responses in tuberculosis patients. Microb. Pathog..

[15] Alam K., Ghousunnissa S., Nair S., Valluri V.L., Mukhopadhyay S. (2010). Glutathione-Redox Balance Regulates c-rel–Driven IL-12 Production in Macrophages: Possible Implications in Antituberculosis Immunotherapy. J. Immunol..

[16] Yew W.W., Leung C.C., Zhang Y. (2017). Oxidative stress and TB outcomes in patients with diabetes mellitus?. J. Antimicrob. Chemother..

[17] Ganatra S.R., Bucşan A.N., Alvarez X., Kumar S., Chatterjee A., Quezada M., Fish A.I., Singh D.K., Singh B., Sharan R. (2020). Antiretroviral therapy does not reduce tuberculosis reactivation in a tuberculosis-HIV coinfection model. J. Clin. Investig..

[18] Leung C.C., Lam T.H., Chan W.M., Yew W.W., Ho K.S., Leung G.M., Law W.S., Tam C.M., Chan C.K., Chang K.C. (2008). Diabetic Control and Risk of Tuberculosis: A Cohort Study. Am. J. Epidemiol..

[19] Allen M., Bailey C., Cahatol I., Dodge L., Yim J., Kassissa C., Luong J., Kasko S., Pandya S., Venketaraman V. (2015). Mechanisms of Control of *Mycobacterium tuberculosis* by NK Cells: Role of Glutathione. Front. Immunol..

[20] Morris D., Ly J., Chi P.-T., Daliva J., Nguyen T., Soofer C., Chen Y.C., Lagman M., Venketaraman V. (2014). Glutathione synthesis is compromised in erythrocytes from individuals with HIV. Front. Pharmacol..

[21] Lutchmansingh F.K., Hsu J.W., Bennett F.I., Badaloo A., McFarlane-Anderson N., Gordon-Strachan G.M., Wright-Pascoe R.A., Jahoor F., Boyne M.S. (2018). Glutathione metabolism in type 2 diabetes and its relationship with microvascular complications and glycemia. PLoS ONE.

[22] Islamoglu H., Cao R., Teskey G., Gyurjian K., Lucar S., Fraix M.P., Sathananthan A., Chan J.K., Venketaraman V. (2018). Effects of ReadiSorb L-GSH in Altering Granulomatous Responses against *Mycobacterium tuberculosis* Infection. J. Clin. Med..

[23] Ly J., Lagman M., Saing T., Singh M.K., Tudela E.V., Morris D., Anderson J., Daliva J., Ochoa C., Patel N. (2015). Liposomal Glutathione Supplementation Restores T_H_1 Cytokine Response to *Mycobacterium tuberculosis* Infection in HIV-Infected Individuals. J. Interferon Cytokine Res..

[24] To K., Cao R., Yegiazaryan A., Owens J., Nguyen T., Sasaninia K., Vaughn C., Singh M., Truong E., Medina A. (2021). Effects of Oral Liposomal Glutathione in Altering the Immune Responses against *Mycobacterium tuberculosis* and the *Mycobacterium bovis* BCG Strain in Individuals With Type 2 Diabetes. Front. Cell. Infect. Microbiol..

[25] Lagman M., Ly J., Saing T., Singh M.K., Tudela E.V., Morris D., Chi P.-T., Ochoa C., Sathananthan A., Venketaraman V. (2015). Investigating the Causes for Decreased Levels of Glutathione in Individuals with Type II Diabetes. PLoS ONE.

[26] Subbian S., Tsenova L., Yang G., O’Brien P., Parsons S., Peixoto B., Taylor L., Fallows D., Kaplan G. (2011). Chronic pulmonary cavitary tuberculosis in rabbits: A failed host immune response. Open Biol..

[27] Subbian S., Pandey R., Soteropoulos P., Rodriguez G.M. (2015). Vaccination with an Attenuated Ferritin Mutant Protects Mice against Virulent *Mycobacterium tuberculosis*. J. Immunol. Res..

[28] Muñoz-Elías E.J., Timm J., Botha T., Chan W.T., Gomez J.E., McKinney J.D. (2005). Replication Dynamics of *Mycobacterium tuberculosis* in Chronically Infected Mice. Infect. Immun..

[29] Tsenova L., Fallows D., Kolloli A., Singh P., O’Brien P., Kushner N., Kaplan G., Subbian S. (2020). Inoculum size and traits of the infecting clinical strain define the protection level against *Mycobacterium tuberculosis* infection in a rabbit model. Eur. J. Immunol..

[30] Kolloli A., Kumar R., Singh P., Narang A., Kaplan G., Sigal A., Subbian S. (2021). Aggregation state of *Mycobacterium tuberculosis* impacts host immunity and augments pulmonary disease pathology. Commun. Biol..

[31] Subbian S., Tsenova L., Holloway J., Peixoto B., O’Brien P., Dartois V., Khetani V., Zeldis J.B., Kaplan G. (2016). Adjunctive phosphodiesterase-4 inhibitor therapy improves antibiotic response to pulmonary tuberculosis in a rabbit model. EBioMedicine.

[32] Narayanankutty A., Job J.T., Narayanankutty V. (2019). Glutathione, an Antioxidant Tripeptide: Dual Roles in Carcinogenesis and Chemoprevention. Curr. Protein Pept. Sci..

[33] Pal R., Ansari M.A., Hameed S., Fatima Z. (2016). Diabetes Mellitus as Hub for Tuberculosis Infection: A Snapshot. Int. J. Chronic Dis..

[34] Guerra C., Johal K., Morris D., Moreno S., Alvarado O., Gray D., Tanzil M., Pearce D., Venketaraman V. (2012). Control of *Mycobacterium tuberculosis* growth by activated natural killer cells. Clin. Exp. Immunol..

[35] Dayaram Y.K., Talaue M.T., Connell N.D., Venketaraman V. (2006). Characterization of a Glutathione Metabolic Mutant of *Mycobacterium tuberculosis* and Its Resistance to Glutathione and Nitrosoglutathione. J. Bacteriol..

[36] Cao R., Kolloli A., Kumar R., Owens J., Sasaninia K., Vaughn C., Singh M., Truong E., Kachour N., Beever A. (2021). Effects of Glutathione Diminishment on the Immune Responses against *Mycobacterium tuberculosis* Infection. Appl. Sci..

[37] Morris D., Guerra C., Khurasany M., Guilford F., Saviola B., Huang Y., Venketaraman V. (2013). Glutathione Supplementation Improves Macrophage Functions in HIV. J. Interferon Cytokine Res..

[38] Rao M., Ippolito G., Mfinanga S., Ntoumi F., Yeboah-Manu D., Vilaplana C., Zumla A., Maeurer M. (2019). Latent TB Infection (LTBI)—*Mycobacterium tuberculosis* pathogenesis and the dynamics of the granuloma battleground. Int. J. Infect. Dis..

[39] Warsinske H.C., DiFazio R.M., Linderman J.J., Flynn J.L., Kirschner D.E. (2017). Identifying mechanisms driving formation of granuloma-associated fibrosis during *Mycobacterium tuberculosis* infection. J. Theor. Biol..

[40] Crouser E.D., White P., Caceres E.G., Julian M.W., Papp A.C., Locke L.W., Sadee W., Schlesinger L.S. (2017). A Novel in vitro Human Granuloma Model of Sarcoidosis and Latent Tuberculosis Infection. Am. J. Respir. Cell. Mol. Biol..

[41] Venketaraman V., Dayaram Y.K., Amin A.G., Ngo R., Green R.M., Talaue M.T., Mann J., Connell N.D. (2003). Role of glutathione in macrophage control of mycobacteria. Infect. Immun..

[42] Venketaraman V., Dayaram Y.K., Talaue M.T., Connell N.D. (2005). Glutathione and nitrosoglutathione in macrophage defense against *Mycobacterium tuberculosis*. Infect. Immun..

[43] Henry C.J., Ornelles D.A., Mitchell L.M., Brzoza-Lewis K.L., Hiltbold E.M. (2008). IL-12 Produced by Dendritic Cells Augments CD8^+^ T Cell Activation through the Production of the Chemokines CCL1 and CCL17. J. Immunol..

[44] Nishikomori R., Ehrhardt R.O., Strober W. (2000). T Helper Type 2 Cell Differentiation Occurs in the Presence of Interleukin 12 Receptor β2 Chain Expression and Signaling. J. Exp. Med..

[45] Wang T., Hu Y., Wangkahart E., Liu F., Wang A., Zahran E., Maisey K., Liu M., Xu Q., Imarai M. (2018). Interleukin (IL)-2 Is a Key Regulator of T Helper 1 and T Helper 2 Cytokine Expression in Fish: Functional Characterization of Two Divergent IL2 Paralogs in Salmonids. Front. Immunol..

[46] Eyerich K., Dimartino V., Cavani A. (2017). IL-17 and IL-22 in immunity: Driving protection and pathology. Eur. J. Immunol..

[47] Beringer A., Noack M., Miossec P. (2016). IL-17 in Chronic Inflammation: From Discovery to Targeting. Trends Mol. Med..

[48] Wang X., Wong K., Ouyang W., Rutz S. (2019). Targeting IL-10 Family Cytokines for the Treatment of Human Diseases. Cold Spring Harb. Perspect. Biol..

[49] Batlle E., Massagué J. (2019). Transforming Growth Factor-β Signaling in Immunity and Cancer. Immunity.

[50] Didion S. (2017). Cellular and Oxidative Mechanisms Associated with Interleukin-6 Signaling in the Vasculature. Int. J. Mol. Sci..

[51] Lin P.L., Flynn J.L. (2010). Understanding Latent Tuberculosis: A Moving Target. J. Immunol..

[52] Evans S., Butler J.R., Mattila J.T., Kirschner D.E. (2020). Systems biology predicts that fibrosis in tuberculous granulomas may arise through macrophage-to-myofibroblast transformation. PLoS Comput. Biol..

[53] Pagán A.J., Ramakrishnan L. (2018). The Formation and Function of Granulomas. Annu. Rev. Immunol..

[54] Bonham C.A., Strek M.E., Patterson K.C. (2016). From granuloma to fibrosis: Sarcoidosis associated pulmonary fibrosis. Curr. Opin. Pulm. Med..

[55] Prokop S., Heppner F.L., Goebel H.H., Stenzel W. (2011). M2 Polarized Macrophages and Giant Cells Contribute to Myofibrosis in Neuromuscular Sarcoidosis. Am. J. Pathol..

[56] Al Shammari B., Shiomi T., Tezera L., Bielecka M.K., Workman V., Sathyamoorthy T., Mauri F., Jayasinghe S., Robertson B.D., D’Armiento J. (2015). The extracellular matrix regulates granuloma necrosis in tuberculosis. J. Infect. Dis..

[57] Young D. (2009). Animal models of tuberculosis. Eur. J. Immunol..

[58] Ravimohan S., Kornfeld H., Weissman D., Bisson G.P. (2018). Tuberculosis and lung damage: From epidemiology to pathophysiology. Eur. Respir. Rev..

[59] Pérez-Torres I., Guarner-Lans V., Rubio-Ruiz M.E. (2017). Reductive Stress in Inflammation-Associated Diseases and the Pro-Oxidant Effect of Antioxidant Agents. Int. J. Mol. Sci..

[60] Tan K.S., Lee K.-O., Low K.C., Gamage A.M., Liu Y., Tan G.-Y.G., Koh H.Q.V., Alonso S., Gan Y.-H. (2012). Glutathione deficiency in type 2 diabetes impairs cytokine responses and control of intracellular bacteria. J. Clin. Investig..

